# A case report and literature review of sigmoid volvulus in children

**DOI:** 10.1097/MD.0000000000009434

**Published:** 2017-12-29

**Authors:** Po-Hsiung Chang, Chin-Ming Jeng, Der-Fang Chen, Lung-Huang Lin

**Affiliations:** a School of Medicine, Taipei Medical University, Taipei City; b Department of Radiology; c Department of Surgery; d Department of Pediatrics, Cathay General Hospital, Taipei; e School of Medicine, Fu Jen Catholic University, New Taipei City, Taiwan.

**Keywords:** abdominal distention, children, sigmoid volvulus

## Abstract

**Rationale:**

Sigmoid volvulus (SV) is an exceptionally rare but potentially life-threatening condition in children.

**Chief complaint:**

Abdominal distention for 1 week.

**Diagnoses:**

Sigmoid volvulus.

**Patient concerns:**

We present a case of a 12-year-old boy with mechanical ileus who was finally confirmed to have SV with the combination of abdominal plain film, sonography, and computed tomography (CT) with the finding of mesenteric artery rotation.

**Interventions:**

Because bowel obstruction was suspected, abdominal plain film, sonography, and CT were performed. The abdominal CT demonstrated whirlpool sign with torsion of the sigmoid vessels. In addition, lower gastrointestinal filling study showed that the contrast medium could only reach the upper descending colon. Therefore, he received laparotomy with mesosigmoidoplasty for detorsion of the sigmoid.

**Outcomes:**

The postoperative recovery was smooth under empirical antibiotic treatment with cefazolin. A follow-up lower gastrointestinal series on the seventh day of admission showed no obstruction compared with the previous series. He was finally discharged in a stable condition 8 days after admission.

**Lessons:**

SV is a congenital anomaly and an uncommon diagnosis in children. Nevertheless, case series and case reports of SV are becoming more prevalent in the literature. Failure to recognize SV may result in life-threatening complications such as sigmoid gangrene/perforation, peritonitis, sepsis, and death. Thus, if the children have persistent and recurrent abdominal distention, abdominal pain, and vomiting, physicians should consider SV as a “do not miss diagnosis” in the differential diagnosis.

## 1 Introduction

The prevalence of sigmoid volvulus (SV) varies widely in the world, with some places called “volvulus belts” where high-fiber diets are the norm.^[1]^ SV is in majority among males between 40 and 80 years of age. It is more common among Eastern countries and accounts for about 20% to 50% cases of colonic obstructions. However, SV is responsible for about 2% to 5% of colonic obstruction cases in the Western world.^[2]^ In the United States, the annual incidence of SV has been reported to be 1.67 in 100,000 persons.^[2]^ While SV is common in the elderly, it is rare in children and can resolve spontaneously. Because of this, the diagnosis is usually missed or delayed.^[3]^ In Salas et al,^[4]^ there are 63 cases of SV in children in a case series from 1941 to 2000. Besides, Smith et al^[5]^ reported 48 cases of SV in children in a comprehensive review of literature in 1990. The majority of patients with SV present with the insidious onset of slowly progressive nausea, abdominal pain and distention, and vomiting after the onset of pain for several days. It is often relieved by the passage of stool or flatus, and therefore constipation is a common misdiagnosis. Due to its insidious presentation, most patients present 3 to 4 days after the onset of symptoms. If SV is untreated, it may progress to ischemic colon, hemorrhagic infarction, and even death; as these consequences are potentially life-threatening, physicians should consider SV in the differential for patients presenting with acute or recurrent abdominal pain and bowel obstruction. Herein, we present the case of a 12-year-old boy with persistent abdominal distension for 1 week diagnosed of SV.

## 2 Method

This case report was approved by the Institutional Review Board of Cathay General Hospital, Taipei, Taiwan. Informed consent was obtained from the patients and parents.

### 2.1 Consent

Written informed consent was obtained from the patient and parents for publication of this case report and any accompanying images.

### 2.2 Case presentation [patient or subject (under study)]

**Figure 1:**
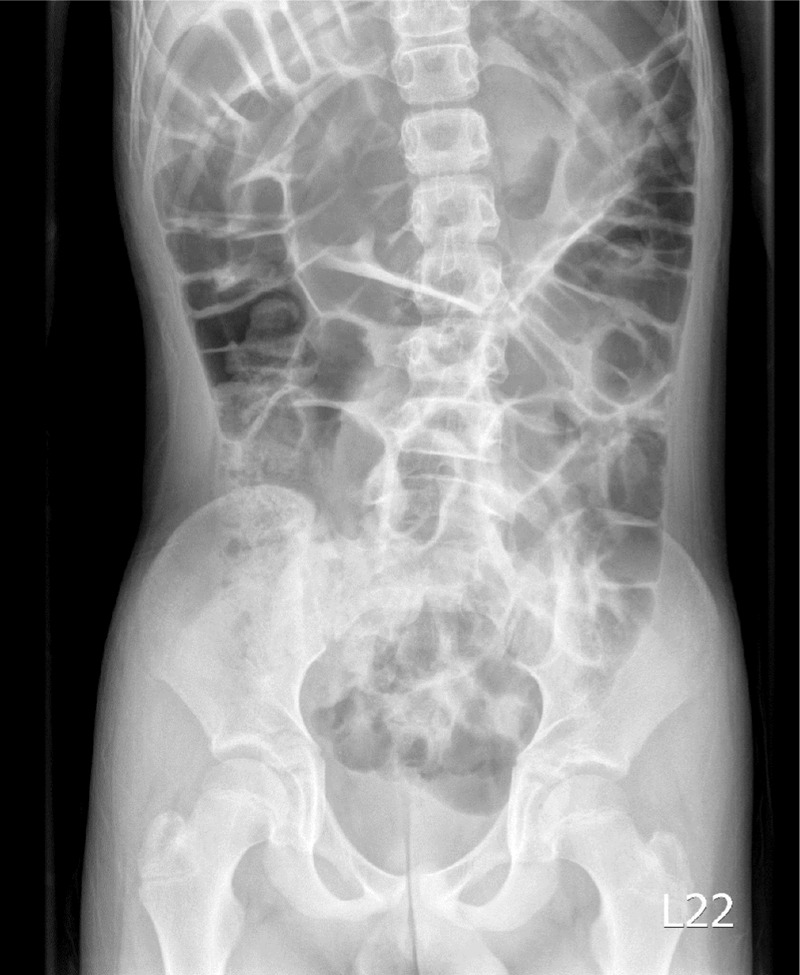
Radiograph demonstrating a greatly dilated sigmoid that almost filled the entire abdomen 1 day before admission.

This 12-year-old boy presented with autism and a history of hearing impairment in his right ear. He had been well until about 1 week before this presentation, when nausea, persistent abdominal distention, poor appetite, and reduced activity were noted by his parents. He did not have a fever or diarrhea. His family brought him to the Emergency Department of Cathay General Hospital on September 21, 2016. A physical examination at admission revealed a massively distended abdomen without muscle guarding or rebounding pain. The initial laboratory tests showed a white blood cell count of 9.88 × 10^3^ cells/mm^3^ [normal reference (NR): 4–10 × 10^3^/μL] with elevated segments (86.4%) [NR: 40–75%], normocytic anemia (Hb: 13.9 g/dL; MCV: 82 fL) [NR: Hb: 14–18 g/dL; MCV: 81–97 fL] and a C-reactive protein (CRP) level of 0.284 mg/dL [NR: 0.01–0.5 mg/dL]. Abdominal plain film revealed severe colonic distention with gas over his abdomen suggesting ileus (Fig. 1). He was then admitted under the tentative diagnosis of abdominal distention with unknown cause, and intravenous metoclopramide was initially given empirically.

**Figure 2:**
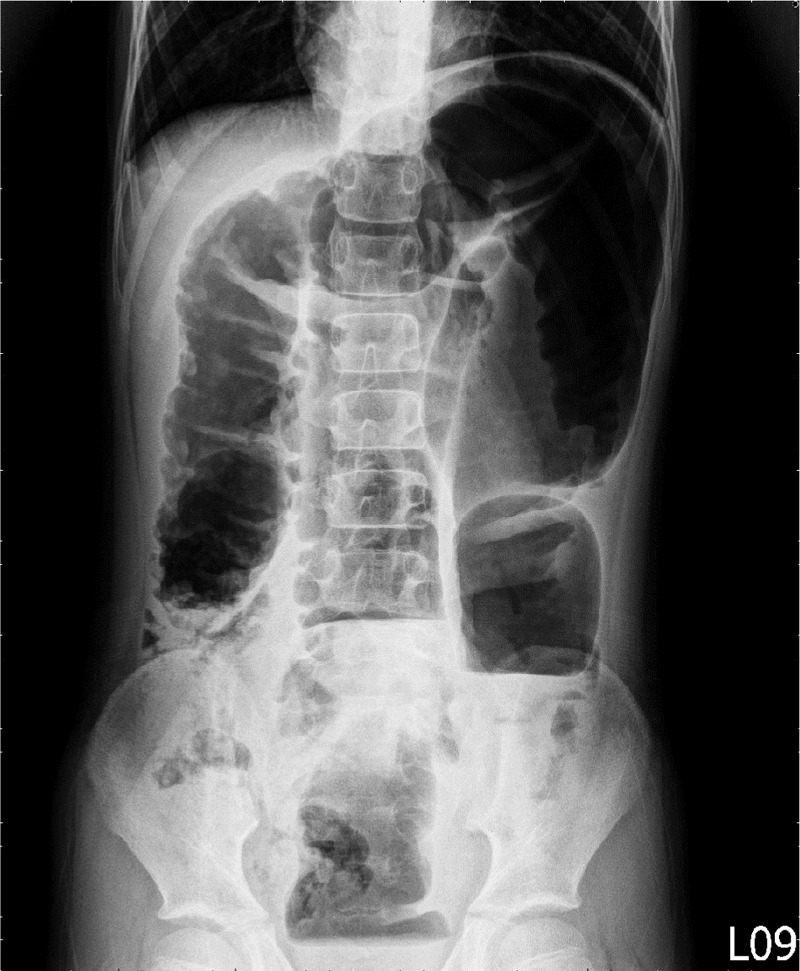
KUB showing an inverted “U” sign on the second day of admission.

To rule out acute gastroenteritis or infectious colitis, we tested for rotavirus antigen which showed negative results, and a stool culture/analysis revealed no significant findings with no parasite ova or occult blood. Hirschsprung disease (HD) was actually not in our consideration. According to our patient's history, he did not fail to pass the meconium within 48 hours of delivery which is typical HD symptom. There were no vomiting green or brown substance, bloody diarrhea, swollen belly, excessive intestinal gas, and explosive stools after a doctor inserts a finger into the rectum before his age of 10. Therefore, HD could be excluded (Fig. 2).

**Figure 3:**
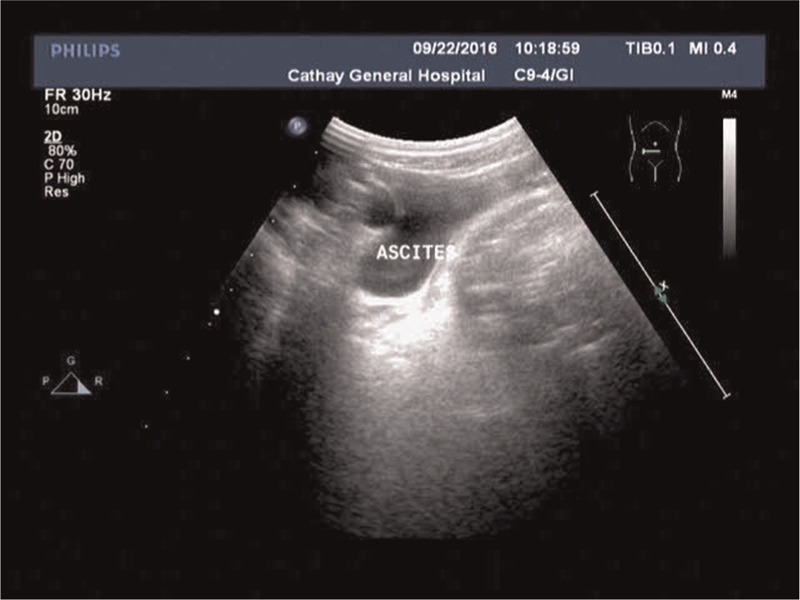
Ultrasound revealed ascites and a greatly dilated sigmoid colon.

**Figure 4:**
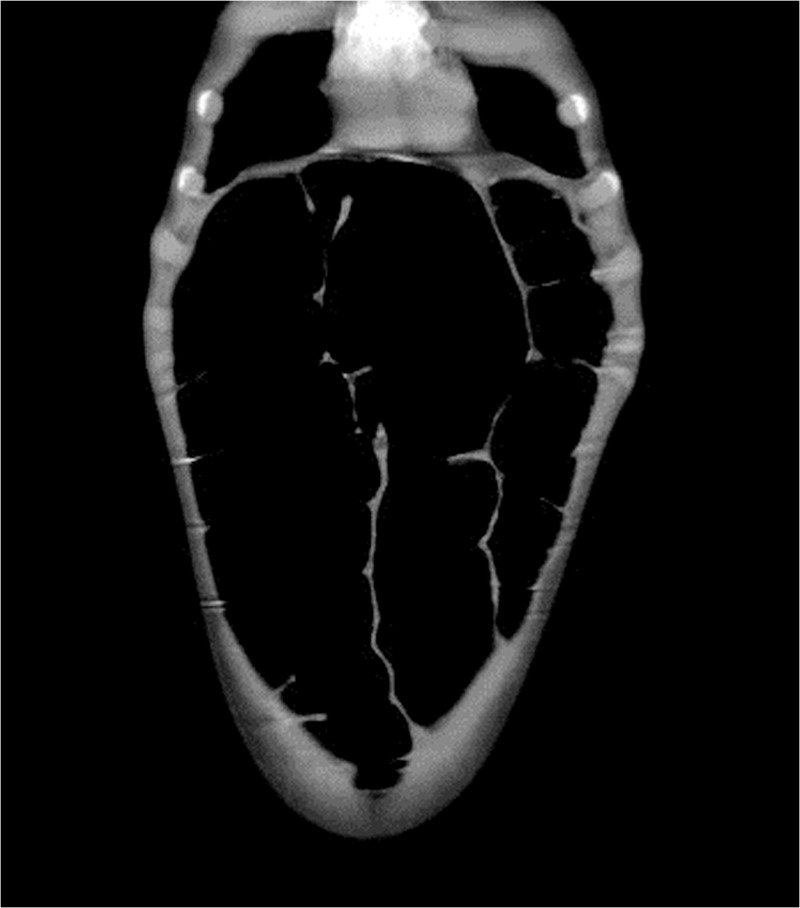
Sigmoid colon overlapping the liver and extended cephalad to the transverse colon in abdominal CT.

**Figure 5:**
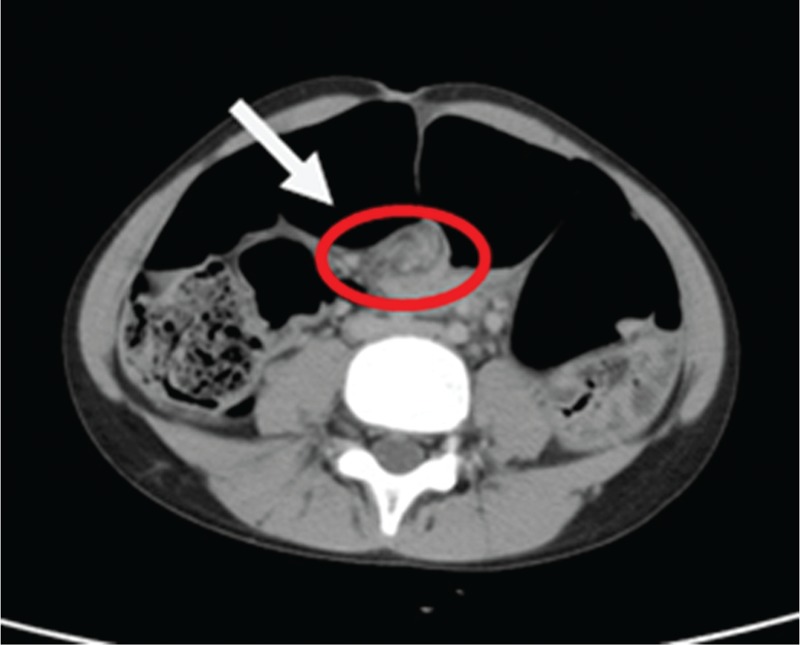
Whirlpool sign (arrow) with torsion of the sigmoid vessels in abdominal CT.

**Figure 6:**
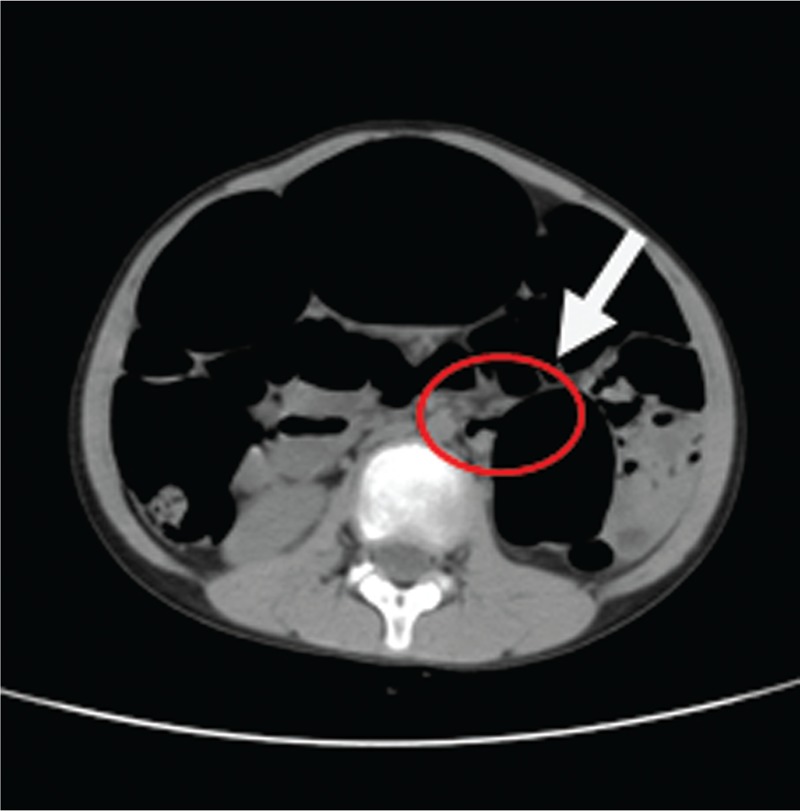
Transition point with an abrupt reduction in bowel caliber seen as a “beak” or “ace of spades” sign in abdominal CT (arrow).

**Figure 7:**
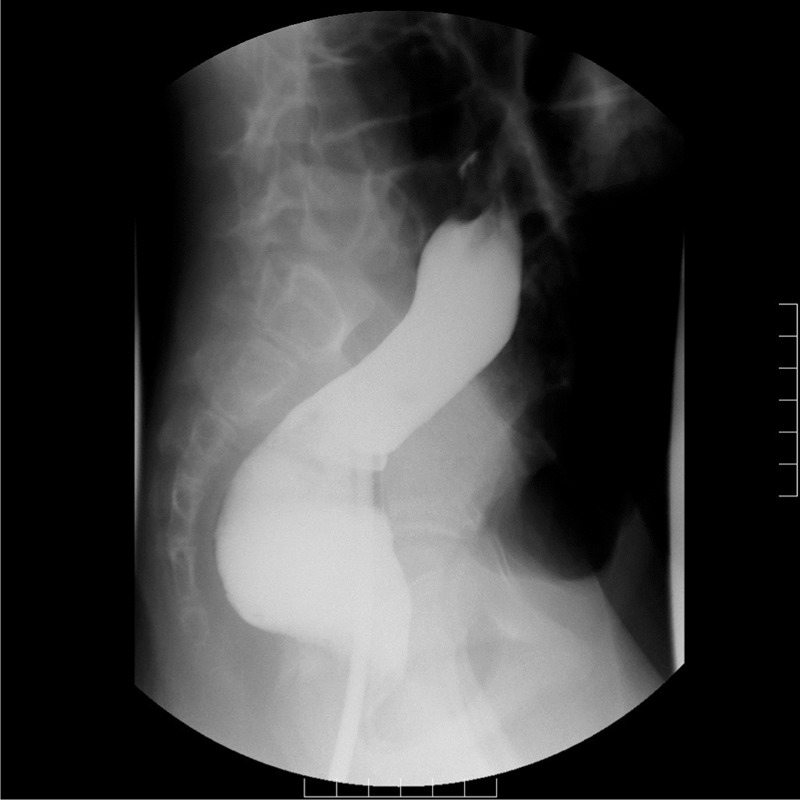
Lower gastrointestinal filling study showed that the contrast medium could only reach the upper descending colon.

**Figure 8:**
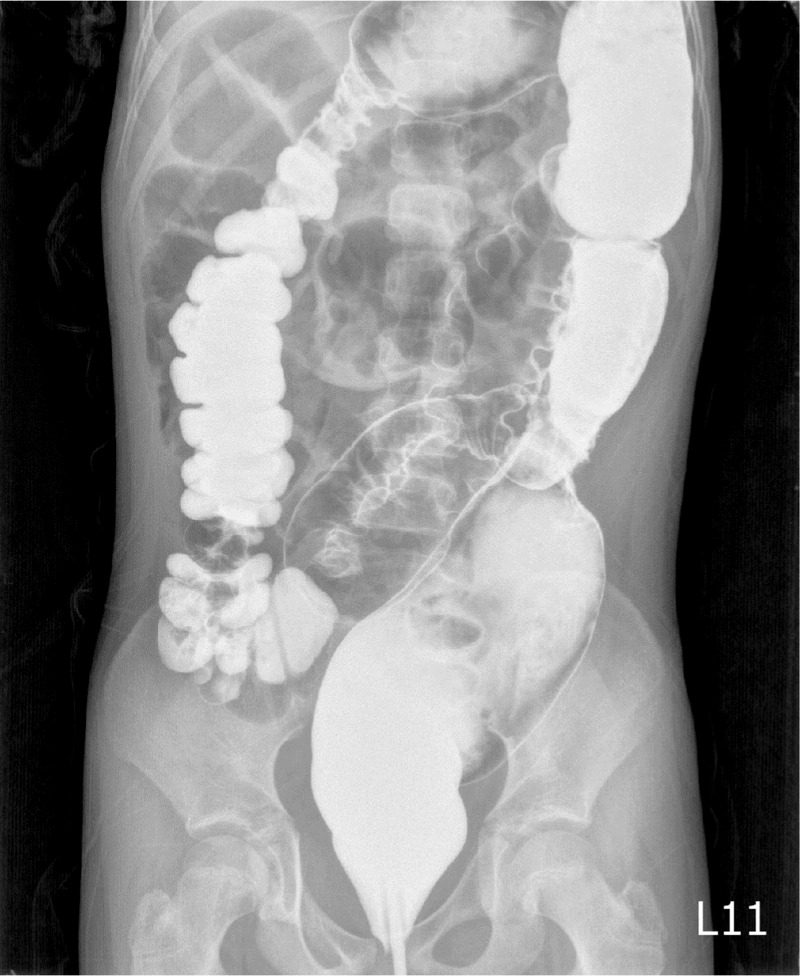
A second lower gastrointestinal filling study was arranged 3 days later after conservative treatment, which showed that the contrast medium passed smoothly from the colon to the terminal ileum with normal caliber.

However, there was no clinical improvement even under parental metoclopramide and Bisacodyl spp. Abdominal ultrasound showed ascites and meteorism (Fig. 3). The imaging findings combined with the persistent abdominal distention suggested mechanical ileus. We arranged computed tomography (CT) on the second day after admission which showed marked gaseous distention of the whole colon, and especially the sigmoid colon (with a maximum diameter of up to 7.3 cm). The small bowel did not seem to be dilated with fluid collection in the lower pelvic cavity; however, mesenteric artery rotation was noted (Figs. 4 and 5). We also discovered a transition point with an abrupt reduction in bowel caliber seen as a “beak” or “ace of spades sign in abdominal CT (Fig. 6). A lower gastrointestinal study with a barium enema yielded marked tapering and luminal narrowing at the sigmoid colon and poor barium passage proximally, indicating nearly complete obstruction of the sigmoid colon which confirmed sigmoid colon volvulus (Fig. 7). He then received laparotomy with mesosigmoidoplasty for detorsion of the sigmoid on the third day of admission. During laparotomy, insertion of a rectal tube with suction was performed, and the bowel distension resolved completely. In addition, 270° counterclockwise torsion of the sigmoid colon was confirmed during the operation. One day after surgery, a lower gastrointestinal series showed a redundant sigmoid colon (Fig. 8). The postoperative recovery was smooth under empirical antibiotic treatment with cefazolin. A follow-up lower gastrointestinal series on the seventh day of admission showed no obstruction compared with the previous series. He was finally discharged in a stable condition 8 days after admission. With his following up in our outpatient department, he recovered well and experienced no adverse events in the 3 months postsurgery.

## 3 Discussion

Volvulus can develop in any portion of the large bowel; however, it is most common in the sigmoid colon because of the mesenteric anatomy. In the pediatric surgical practice, SV remains a rare incidence. Only few isolated case reports and case series have been reported. Salas et al^[4]^ reported 63 cases of SV in children (median age: 7 years old) and males outnumbered females by a ratio of 3.5:1 from 1941 to 2000. Mellor and Drake in 1994^[6]^ reported only 14 cases of colonic volvulus in children, with 10 cases of SV. SV occurs when a redundant sigmoid loop rotates around its narrow and elongated mesentery leading to venous and arterial obstruction of the affected segment followed by rapid distention of the closed loop. If untreated, it can result in hemorrhagic infarction, perforation, septic shock, and death.^[7]^ Obstruction of the intestinal lumen and impairment of vascular perfusion occurs when the degree of torsion exceeds 180° and 360°, respectively.^[8]^ This is consistent with our patient, where his SV was twisted at about 270° so that the barium enema could not pass from his rectum to his descending colon.

The diagnosis of SV is made from a detailed history, physical examination, and careful interpretation of plain abdominal films. The diagnostic findings of SV include a whirlpool pattern caused by the dilated sigmoid colon around its mesocolon and vessels, and a bird-beak appearance of the afferent and efferent colonic segments. However, abdominal X-rays in children are often nonspecific and are less useful in distinguishing volvulus from other disorders.^[5]^ In addition, typical imaging features have been reported to be absent in one-fourth of CT scans, as with our patient. A gas pattern is often not helpful diagnostically, and the single U-shaped sigmoid loop characteristic of SV is absent in adults.^[4]^ Other supportive features of SV include the absence of rectal gas, apparent separation of the sigmoid walls by adjacent mesenteric fat due to incomplete twisting or folding (split wall sign), and 2 crossing sigmoid transition points projecting from a single location. In addition, when a dilated, twisted sigmoid colon is seen in association with a proximal obstruction, the findings are diagnostic; the diagnosis of SV cannot be made from plain films unless this configuration is seen.^[9]^

In patients with no evidence of peritonitis or ischemic bowel, treatment should start with resuscitation and detorsion of the SV, which can be accomplished by sigmoidoscopy and concomitant rectal tube placement. A barium enema, which is a treatment option for SV, can also be of diagnostic value. As in adults, a barium enema increases radiographic diagnostic sensitivity in pediatric patients (71–82%).^[10]^ All nonoperative modalities for decompression carry the risk of perforation. However, the management of SV is controversial. Some experts recommend that endoscopy be reserved for patients who are not candidates for definitive surgical therapy, while others recommend surgery be reserved for patients in whom sigmoidoscopic reduction is unsuccessful, since approximately 40% to 50% of patients with SV will not experience recurrence.

The goal of SV treatment is to reduce the SV and prevent recurrent episodes. In our case, on account of our patient's symptoms presenting not so urgent and no severe abdominal pain initially, we did not take prompt notice to the surgeon until 48 hours of admission with the abdominal plain film suggesting ileus. Medical treatment with intravenous metoclopramide and fluid infusion were instead preferred. However, we perform immediate laparotomy when endoscopic detorsion is unsuccessful or in patients with signs and symptoms suggestive of peritonitis at our hospital. The definitive treatment for SV is sigmoidectomy, either with primary anastomosis or colostomy. Recurrence is common when detorsion is attempted without resection (operative 25%, nonoperative 35%),^[4,11]^ whereas recurrent SV after sigmoidectomy has never been reported,^[3]^ In conclusion, SV is a congenital anomaly and an uncommon diagnosis in children. Nevertheless, case series and case reports of SV are becoming more prevalent in the literature. Failure to recognize SV may result in life-threatening complications such as sigmoid gangrene/perforation, peritonitis, sepsis, and death.^[4]^ Thus, if the children have persistent and recurrent abdominal distention, abdominal pain, and vomiting, physicians should consider SV as a “do not miss diagnosis” in the differential diagnosis.
